# Coronavirus disease 2019 (COVID-19): an evidence map of medical literature

**DOI:** 10.1186/s12874-020-01059-y

**Published:** 2020-07-02

**Authors:** Nan Liu, Marcel Lucas Chee, Chenglin Niu, Pin Pin Pek, Fahad Javaid Siddiqui, John Pastor Ansah, David Bruce Matchar, Sean Shao Wei Lam, Hairil Rizal Abdullah, Angelique Chan, Rahul Malhotra, Nicholas Graves, Mariko Siyue Koh, Sungwon Yoon, Andrew Fu Wah Ho, Daniel Shu Wei Ting, Jenny Guek Hong Low, Marcus Eng Hock Ong

**Affiliations:** 1 Duke-NUS Medical School, National University of Singapore, Singapore, Singapore; 2 Health Services Research Centre, Singapore Health Services, Singapore, Singapore; 3 Faculty of Medicine, Nursing and Health Sciences, Monash University, Melbourne, Australia; 4 Department of Emergency Medicine, Singapore General Hospital, Singapore, Singapore; 5 Department of Medicine, Duke University School of Medicine, Durham, NC USA; 6 Department of Anaesthesiology, Singapore General Hospital, Singapore, Singapore; 7 Department of Respiratory and Critical Care Medicine, Singapore General Hospital, Singapore, Singapore; 8 Singapore National Eye Centre, Singapore, Singapore; 9 Department of Infectious Diseases, Singapore General Hospital, Singapore, Singapore

**Keywords:** COVID-19, SARS-CoV-2, Coronavirus, Evidence gap map, Review

## Abstract

**Background:**

Since the beginning of the COVID-19 outbreak in December 2019, a substantial body of COVID-19 medical literature has been generated. As of June 2020, gaps and longitudinal trends in the COVID-19 medical literature remain unidentified, despite potential benefits for research prioritisation and policy setting in both the COVID-19 pandemic and future large-scale public health crises.

**Methods:**

In this paper, we searched PubMed and Embase for medical literature on COVID-19 between 1 January and 24 March 2020. We characterised the growth of the early COVID-19 medical literature using evidence maps and bibliometric analyses to elicit cross-sectional and longitudinal trends and systematically identify gaps.

**Results:**

The early COVID-19 medical literature originated primarily from Asia and focused mainly on clinical features and diagnosis of the disease. Many areas of potential research remain underexplored, such as mental health, the use of novel technologies and artificial intelligence, pathophysiology of COVID-19 within different body systems, and indirect effects of COVID-19 on the care of non-COVID-19 patients. Few articles involved research collaboration at the international level (24.7%). The median submission-to-publication duration was 8 days (interquartile range: 4–16).

**Conclusions:**

Although in its early phase, COVID-19 research has generated a large volume of publications. However, there are still knowledge gaps yet to be filled and areas for improvement for the global research community. Our analysis of early COVID-19 research may be valuable in informing research prioritisation and policy planning both in the current COVID-19 pandemic and similar global health crises.

## Background

On 11 March 2020, the Director-General of the World Health Organization (WHO), Dr. Tedros Adhanom Ghebreyesus, declared coronavirus disease 2019 (COVID-19) a pandemic, approximately 11 weeks after the first detected case of pneumonia of unknown aetiology in Wuhan, China was reported to the WHO Country Office in China on 31 December 2019 [1]. As of 14 June 2020, 7,625,883 cases of COVID-19 have been reported in 209 countries and territories, including 425,931 deaths [2]. COVID-19 is caused by the novel betacoronavirus SARS-CoV-2, which is genetically similar to but distinct [3] from other coronaviruses responsible for global outbreaks such as SARS-CoV-1 [4, 5] and MERS-CoV [6].

COVID-19 has attracted tremendous interest from researchers and clinicians worldwide, resulting in an appreciable body of COVID-19 literature being published in a relatively short period. Given the urgent need for evidence to support clinical and public health decisions, researchers have begun summarising and analysing the published literature to aggregate current evidence in the form of systematic reviews [7–9] and bibliometric analyses [10–12]. Existing bibliometric analyses [11, 12] have provided overviews of the COVID-19 research landscape. However, they primarily focus on authorship, keywords, and collaboration patterns without identifying gaps in the literature. More recent work includes a living mapping and living systematic review of randomised controlled trials (RCTs) which provides an up-to-date overview of the highest quality evidence on the prevention and treatment of COVID-19 [13]. However, to date, there have been no investigations of gaps for COVID-19 research of all study designs and review types, nor has there been any investigation into longitudinal trends in the literature. Given the large body of literature being generated and the critical role of research in large-scale public health emergencies, an analysis of trends and gaps within the early medical literature would meaningfully inform future direction and priority setting in both the current pandemic and future outbreaks, pandemics, or other rapidly evolving public health crises.

In this paper, we characterised the growth of early medical literature on COVID-19 between 1 January and 24 March 2020 using evidence maps and bibliometric analysis to elicit cross-sectional and longitudinal trends and systematically identify gaps in research within the early phase of the pandemic. Evidence maps in this study were a variant of the Evidence Gap Map (EGM), which is a systematic approach to identifying and describing the research activity in a topic area or policy domain, often through a focused study of systematic reviews [14, 15]. EGMs have been used in a variety of research domains to characterise topic distributions and to inform priority setting in future research [15, 16].

## Methods

### Search strategy and selection criteria

We searched PubMed and Embase databases from 1 January to 24 March 2020 for the keywords “COVID” or “coronavirus” in the title or abstract. We used only these two terms to conduct a broad search that would ensure inclusion of the relevant literature. The search period was chosen on the premise that all articles on COVID-19 were published after the first report of the disease from the Wuhan government on 31 December 2019 [1]. The inclusion criteria for articles were English language, COVID-19 related scientific articles, reviews, and clinical case reports and series. Articles were excluded if they were duplicate articles, editorials, news, commentaries, or opinion pieces.

### Literature selection and data extraction

All extracted literature entries were exported into Microsoft Excel (Office 365) for screening and selection. Between 25 March and 7 April 2020, four reviewers (NL, MLC, CN, and PPP) independently screened the titles, abstracts, and, if ambiguous, full texts for the inclusion of articles. Discrepancies were resolved through discussions among the four reviewers, and in consultation with a fifth reviewer (FJS), to reach a consensus. Subsequently, NL, MLC, CN, and PPP independently conducted information extraction from the included literature. Discrepancies were similarly resolved through discussion among the reviewers and in consultation with FJS.

### Bibliometric analysis

We retrieved basic bibliometric information from online full-text articles of all included articles, including publication date, number of days from submission to online publication, country of the first affiliated institution of the first author, number of countries and institutions represented by the co-authors, and number of co-authors. Citation counts were retrieved from Google Scholar on 7 April 2020. Missing data for the number of days from submission to online publication was common and was treated using pairwise deletion. Other forms of missing data were excluded from the study using listwise deletion.

We performed bibliometric analysis on the retrieved information and presented the results as median (interquartile range [IQR]), proportions, rankings, or other descriptive statistics where appropriate. Additionally, we used the Hersch index (*h*-index) to measure the combination of quality and quantity of research output. An entity (i.e. country or author) has an *h*-index of *h* when it has a maximum of *h* articles with at least *h* citations [17]. Longitudinal trends in the fraction of articles and the fraction of global cases from each continent were analysed and compared using stack plots. Trends in collaboration were analysed using cross-tabulation of international and inter-institutional collaboration; we reported the number (%) of articles that had both international and inter-institutional collaboration, only inter-institutional collaboration, and no international or inter-institutional collaboration.

All bibliometric analyses were conducted using Python version 3.8.0 (Python Software Foundation, Delaware, USA). Data of confirmed COVID-19 cases were obtained from the OurWorldInData.org dataset [18], which aggregates information from daily statistics published by the European Centre for Disease Prevention and Control [2].

### Evidence map analysis

To generate evidence maps, we extracted additional information on the type, topic, and medical speciality of the articles. We categorised original articles into one of the following types: “Observational Research”, “Interventional Research”, “Protocol”, “Research” (basic science research and mathematical or computerised modelling works) or “Case Reports/Series”. Review articles, including narrative and systematic reviews and guidelines, were categorised as “Review”.

Based on the primary focus of the articles, we classified them into one of the following topics: “Basic Science” (articles on basic science or -omics research), “Epidemiology” (including patient risk factors, epidemiological characteristics, and disease trajectory), “Clinical Features and Diagnosis” (articles on all diagnostic elements such as patient signs and symptoms and radiologic or laboratory findings, including molecular diagnosis), “Pathogenesis” (articles reporting on viral mechanisms and disease progression, including immunology), “Treatment” (articles reporting all forms of management, including vaccines), “Public Health” (articles on public health and public policy), “Media” (articles reporting social media related topics), “Technology” (articles reporting the use of technology such as artificial intelligence and smart hardware), and “Health Economics” (articles reporting cost-benefit analysis, quality of life, and related topics). Articles with broad coverage of topics or without a singular focus were classified as “Overview” articles. The topics covered within each overview article were recorded and the distribution of articles covering each topic was presented.

We further classified articles into pre-determined medical specialities. As all articles on COVID-19 are related to infectious and respiratory diseases in some capacity, non-multidisciplinary articles were classified as “Infectious and Respiratory Diseases”, including respiratory medicine and internal medicine as contents of these articles were similar. Multi-disciplinary articles were categorised by their primary speciality into one of the following: “Emergency and Prehospital Care”, “Critical Care” (including intensive care), “Anaesthesiology”, “Ophthalmology”, “Dentistry”, “Cardiology”, “Gastroenterology” (including hepatology), “Nephrology”, “Radiology”, “Pathology”, “Oncology”, “Paediatrics”, “Obstetrics and Gynaecology”, “Psychiatry” (inclusive of mental health-related articles), “Preventive Medicine” (inclusive of public health), and “Family Medicine” (inclusive of general practice-related articles).

To summarize the landscape of current COVID-19 research, several tables were created based on the cross-tabulation of article topics with article types, specialities, and continent of origin. Longitudinal trends across article topics, article types, and specialities were presented using stack plots. The proportion of papers of a certain topic between two continents was compared using the chi-square test. Statistical significance was set at *p* < 0.05.

## Results

**Fig. 1:**
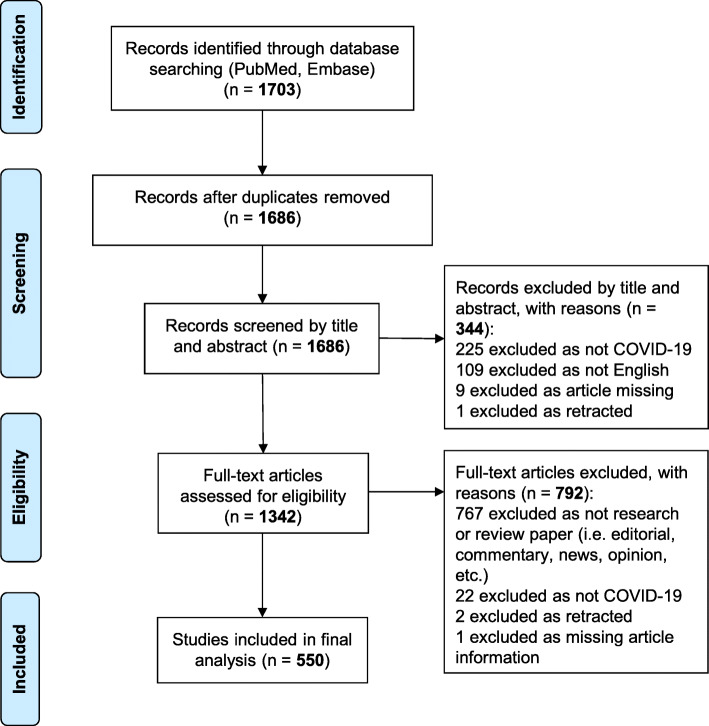
PRISMA flow diagram

The search between 1 January and 24 March 2020 yielded 1703 articles, of which 550 articles (32.3%) met the inclusion criteria for analysis. Figure 1 illustrates the details of the selection process.

### Bibliometric features

Articles were published online over a period of exactly 10 weeks from 14 January to 23 March 2020, with no included articles published on 24 March. The number of new articles per week increased steadily over the first 9 weeks from one in the first week to 119 in the ninth week, dropping to 75 new articles in the final week from 17 to 23 March. Articles for which publication time data was available (*n =* 373, 67.8%) had a median duration of 8 days from submission to online publication (IQR: 4–16). The *h*-index for all 550 included articles was 57, with a cumulative total of 17,450 citations.

**Fig. 2:**
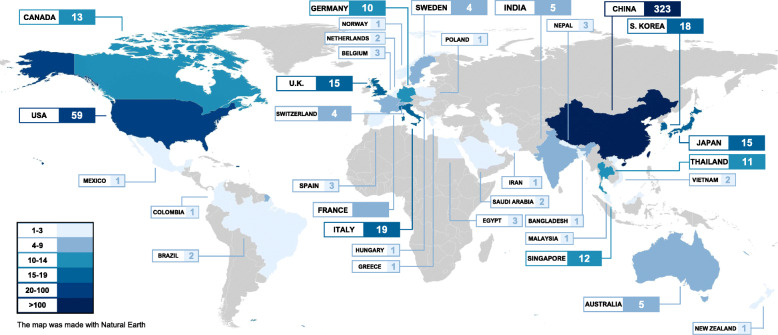
Distribution of articles by country

First authors of the articles were from 33 countries (Fig. 2). China produced the highest output with 323 articles (*h*-index = 48), followed by the United States of America (USA) with 59 articles (*h*-index = 16); 18 of the 33 countries (54.5%) published three or fewer articles.

**Fig. 3:**
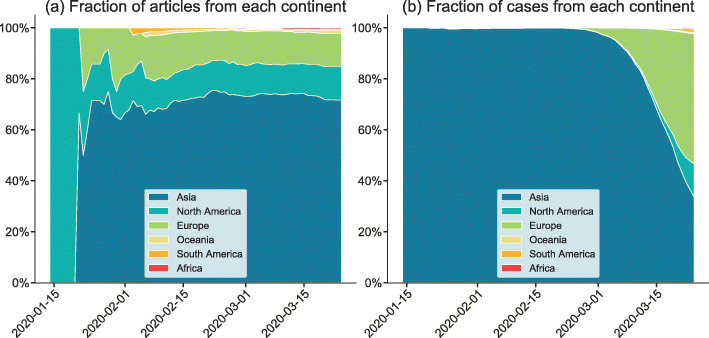
Fractions of **a** articles and **b** cases from each continent from 14 January to 24 March 2020

As of 24 March 2020, articles from Asia (*n* = 394), North America (*n* = 73), and Europe (*n* = 71) represented 71.6, 13.3, and 12.9% of all articles (Fig. 3a), respectively, while confirmed COVID-19 cases from these same continents represented 33.6, 13.1, and 51.0% of 377,261 global cases (Fig. 3b), respectively.

Overall, 183 (33.3%) articles were written by authors from a single institution in one country, 231 (42.0%) articles were written by authors from multiple institutions within one country, while only 136 (24.7%) articles had collaboration between multiple institutions from multiple countries. Articles had a median of six co-authors (IQR: 4–10), including the first author and regardless of institution or country.

### Evidence maps

**Fig. 4:**
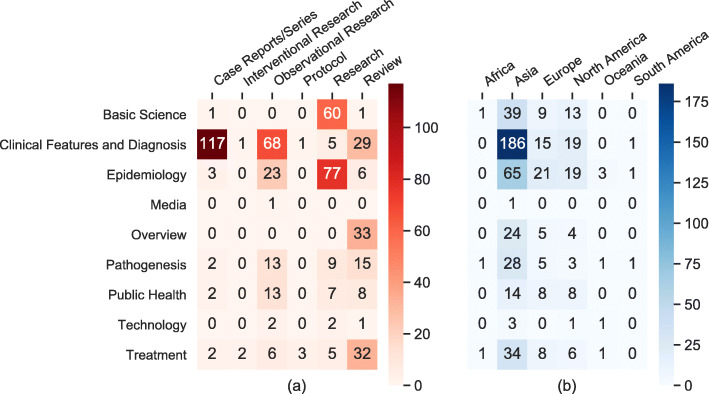
Evidence maps of the distribution of article topics by **a** article type and **b** continent of origin

**Fig. 5:**
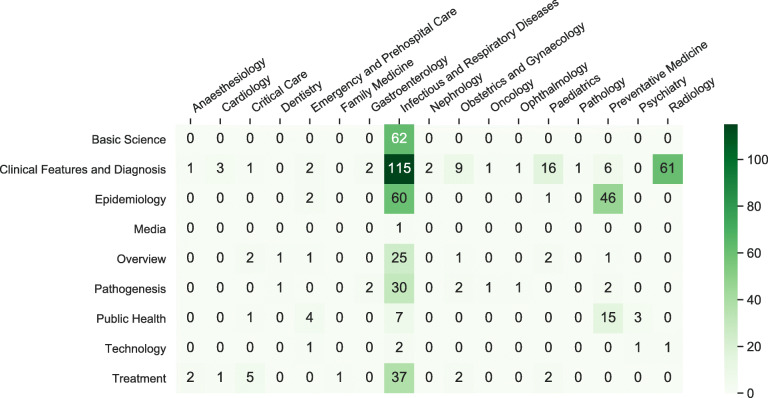
Evidence map of the distribution of article topics by medical speciality

Figures 4 and 5 show the distributions of article topics by article type, continent of origin, and clinical speciality.

According to article topic alone, clinical presentation and diagnosis was the most common (40.2%), followed by epidemiology (19.8%), basic science (11.3%), treatment (9.1%), pathogenicity (7.1%), overview (6.0%), public health (5.5%), technology (0.9%), and media (0.2%); no articles focussed on health economics. By article type alone, general research papers comprising mainly epidemiological or virologic studies were the most common (30.0%), followed by case reports/series (23.1%), observational research (22.9%) and review papers (22.7%). There were few articles on trial protocols (*n* = 4, 0.7%) and interventional research (*n* = 3, 0.5%), of which only one study was conducted on human subjects.

**Fig. 6:**
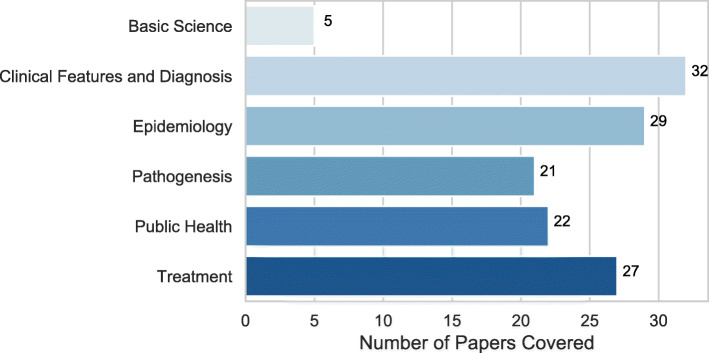
Distribution of topics of overview papers

By article topic and type (Fig. 4a), case reports/series on clinical features and diagnosis (*n* = 117, 21.2%) were the most common, followed by general epidemiological research (*n* = 77, 14.0%) comprising mainly of studies that modelled disease trajectory. Observational research on clinical features and diagnosis (*n* = 68, 12.4%) and general research on basic science (*n* = 60, 10.9%) were also common. There were 33 review papers classified as overview papers, which were further analysed to elucidate the topics covered (Fig. 6). Most overview papers shared similar topics, including clinical features and diagnosis, epidemiology, treatment, pathogenicity, and public health. Only five out of 33 overview papers reported on basic science.

According to the evidence map in Fig. 4b which analysed article topic and continent of origin, papers on clinical features and diagnosis published in Asia were the most common (*n* = 186, 33.8%). Moreover, the proportion of articles reporting on clinical features and diagnosis within articles from Asia (186/394, 47.2%) was significantly higher than the proportion of such articles from North America (19/73, 26.0%), and Europe (15/71, 21.1%), with both *p*-values being less than 0.01. However, for the topic of epidemiology, the proportions of articles from Europe (21/71, 29.6%, *p* = 0.01) and North America (19/73, 26.0%, *p* = 0.07) were higher compared to Asia (65/394, 16.5%).

In terms of the primary speciality of articles, articles on infectious and respiratory diseases predominated (*n* = 339, 61.6%), followed by articles with primary specialities of preventive medicine (*n* = 70, 12.7%), radiology (*n* = 62, 11.2%), and paediatrics (*n* = 21, 3.8%). As shown in Fig. 5, apart from articles on infectious and respiratory diseases, articles on radiological features and diagnosis were the most common (*n* = 61, 11.1%), followed by preventive medicine articles on epidemiology (*n* = 46, 8.4%) and public policy (*n* = 15, 2.7%) and articles on clinical features and diagnosis in paediatric patients (*n* = 16, 2.7%) and obstetric and gynaecological patients (*n* = 9, 1.6%). All other specialities not reported above were lacking in articles, having three or fewer articles on clinical features and diagnosis and, apart from critical care, two or fewer articles on treatment.

**Fig. 7:**
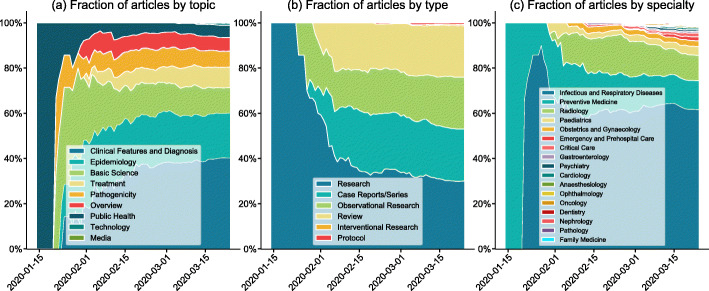
Fractions of articles by **a** topic, **b** type, and **c** speciality from 14 January to 24 March 2020

Longitudinal trends in the fraction of articles by article topic, type, and speciality are shown in Fig. 7a, b, and c, respectively. In the last 5 weeks of the search period, variability in the fraction of articles across article topics, types, and specialities was low with most fractions having an absolute range of less than five percentage points. Exceptions were the fraction of articles with the topic of clinical features and diagnosis (5.9%) and the specialty of radiology (6.3%). Additionally, the combined fraction of article specialities other than the top five most represented specialities increased by an absolute value of 7.0% across the last 5 weeks of the search period (Fig. 7c).

## Discussions

Since the outbreak of COVID-19 in December 2019, more than 1200 relevant articles (as of 24 March 2020) have been published in scientific journals, of which 767 were editorials, commentaries, news, and opinions. Additionally, as of 14 June 2020, 5187 preprints have been archived in medRxiv (4185) and bioRxiv (1002) servers. COVID-19 has garnered research interest faster than any other pandemic in history, possibly due to its high transmissibility [19] fuelled by global interconnectivity; there were fewer articles on SARS and MERS combined within a year after their initial outbreaks than COVID-19 articles within the first 3 months of its discovery [20, 21]. Not surprisingly, one of the first reports [22] on clinical features of patients infected with COVID-19 had attracted 1411 citations within 2 months of publication; its citations reached 7042 by 14 June 2020. Given the large volume and impact of the medical literature in this pandemic, we discuss hereinafter the implications of the present study for the current pandemic as well as relevant insights for future outbreaks, pandemics, or other rapidly evolving public health crises. While the search period of the present study was limited to a relatively early phase in the pandemic comprising the first ten weeks of COVID-19 medical literature, analysis of the cross-sectional features of early articles as well longitiduinal trends within articles' continent of origin, topic, type, and speciality, provided indication of the gaps and trajectory of research across these categories in both the current pandemic and future large-scale public health crises.

A large number of articles focused on epidemiological characteristics and clinical features and diagnosis of COVID-19 patients while there was a dearth of papers that reported findings from RCTs on drugs and treatments; only one clinical trial was conducted on human subjects [23]. This is likely due to insufficient time to design, approve, and execute such trials within the early phase of the pandemic, in contrast to the availability and accessibility of epidemiological and clinical data. Furthermore, we did not search clinical registries such as ClinicalTrials.gov, which indexes clinical trials globally, as the focus of our study was published literature. From the time since our analysis, we observed an increased number of clinical trials and protocols being published and summarised [13].

Another observation is the paucity of technology-related articles in the COVID-19 medical literature. While digital technologies such as artificial intelligence (AI), big data, and the internet have positively impacted public health intervention strategies [24] and shown promising applications in infectious disease contexts [25], few have looked into their applications in COVID-19 research. Despite the straightforward application of AI technology (in particular, deep learning) [26] in analysing medical images, there was only one such study on chest computed tomography (CT) within articles on radiology [27]. Furthermore, despite the high volume of articles on epidemiology, current literature mainly adopted traditional techniques [28] to analyse COVID-19 dynamics [29–31] and forecast trends in the COVID-19 pandemic [32] and its trajectory [33]. Traditional statistical methods rely heavily on underlying assumptions which do not apply to medical data which are multidimensional, dynamic, and highly nonlinear as unpredictable human-environment interactions are involved [25]. More robust and complex modelling has many promising public health applications [34], with similar methods already validated in tuberculosis and gonorrhoea epidemic control [35] and malaria policy [36]. The utility of such models extends to lesser investigated areas in COVID-19 research including social media and public reaction analysis [37, 38]. Beyond the current pandemic, there should be a more extensive and rapid application of advanced technology to research methodology and public health interventions in future public health crises.

As observed in our evidence maps on topic and speciality, apart from articles which focused primarily on infectious and respiratory diseases, there have been substantial works in preventive medicine and radiology, as well as some articles from population-specific specialities such as paediatrics and obstetrics and gynaecology. However, while longitudinal analysis showed a steadily increasing proportion of articles in all other specialites, these specialities were still underrepresented at the end of our search period, leaving obvious gaps in research. For instance, acute kidney and cardiac injury were among the top adverse clinical complications observed in COVID-19 patients with severe disease [22], which should prompt more research on the pathophysiology of disease within specialities like cardiology, nephrology, and critical care where research is sparse. Furthermore, with community clinics, emergency medical services, and emergency departments experiencing a surge in suspected cases of COVID-19, research into the efficient allocation of resources, such as adaptive bed management and operation scheduling, is much needed.

Health care workers have been confronted with unprecedented levels of morbidity and mortality, as well as the constant threat of being exposed to the virus. Quarantine, a common public health measure globally in this pandemic, has significant psychological impacts on those affected [39]. Also, COVID-19 patients and their families can face undue stress as a result of self-blame, fear of transmitting the virus, and uncertainties regarding their health. These are just a few of a multitude of factors that contribute to high psychological stress in persons with and without COVID-19. Regrettably, one of the first research articles exploring the mental health of health care workers in this pandemic was only published at the end of our study’s inclusion period [40]. While other viewpoints [41, 42], guidelines [43], and research [44] on mental health have subsequently surfaced, mental health and psychiatry in all populations remain grossly underrepresented even within the latest literature. Other major clinical settings to explore include but are not limited to neurology [45], anaesthesiology [46], cancer [47], pathology, and geriatrics. Longitudinal trends may indicate an increased focus on these underrepresented specialities as the pandemic progresses. In future pandemics, early attention to multisystem effects and mental health in patients may expedite a more comprehensive understanding of the disease.

It is also important to note that the impact of this pandemic extends beyond COVID-19 patients [48], highlighting the importance of providing high-quality, equal, and continuous care to non-COVID-19 patients. Research should thus be done to quantify the severity and extent to which medical and social care for patients with subacute or chronic conditions have been affected, as well as to uncover other unintended consequences on non-COVID-19 patient care. Such investigations will inform public health response to the current pandemic as well as preparedness and policies to improve outcomes in future public health emergencies.

Our analysis revealed that the submission-to-publication time for COVID-19 articles was much shorter than normal, indicating an accelerated peer-review process. Numerous journals have prioritised COVID-19-related research, giving clinicians, researchers, and the public quicker access to peer-reviewed articles. While this phenomenon is encouraging and a testament to the swift response of the scientific community, this acceleration of the peer-review process during a global health crisis can potentially lead to cursory reviewing and lax publication standards from journals and hasty publication by authors, which may compromise the integrity of research [49]. The undesirable consequences associated with accelerated publication have already surfaced in recent, potentially misleading studies which reported on the positive effects of hydroxychloroquine in COVID-19 [50–52]. Despite the poor study design of these articles [53–55] and potential cardiotoxicity of hydroxychloroquine [56], there was an increase in public demand for hydroxychloroquine following endorsements of its use in COVID-19 treatment [57]. Editors, reviewers, and authors alike have the responsibility of maintaining the integrity of the peer-review process, despite the need for accelerated publication during this crucial period [58].

With the increasing proportion of global cases in Europe, North America, and South America both during and after our period of analysis, the predominance of articles originating from Asia — particularly articles on clinical features and diagnosis — necessitates additional research and evidence from these continents. In the period after our analysis, we observed an increasing number of new articles and preprints from continents other than Asia, although this trend was not captured in our analysis. Cross-country collaborations and initiatives have been limited based on our analysis, despite their essential role in facilitating multi-site clinical trials and patient data sharing. A heartening example is seen in the formation of an international consortium (4 CE) consisting of 96 hospitals across 5 countries [59], in which harmonised electronic health record (EHR) data were analysed locally and converted to a shared aggregate form to analyse and visualise regional differences and global commonalities. The early establishment of international collaborations today will improve global readiness and allow for more rapid data sharing in future global health crises.

With increased understanding of COVID-19, new challenges have also emerged. Unlike SARS, many COVID-19 patients are mildly symptomatic or even asymptomatic [60], which makes screening and identification of cases extremely difficult and leaves the public at risk of infection. The gold standard test based on reverse transcriptase-polymerase chain reaction (RT-PCR) is accurate but is time-consuming, while alternative rapid test kits are fast and make point-of-care diagnosis possible but have shown disappointing performance to date. In general, the current diagnostic tools and technologies are not readily available for large-scale, population-based screening of COVID-19, leaving much room for future research.

Besides those discussed, there are still many unknowns that will require investments from both public and private entities to resolve. For instance, to what extent will a vaccine slow the spread COVID-19? Are repeated infections possible and, if so, how long after the first infection? What is the quality of life among survivors with severe disease? What are effective public health measures at the national and international levels that can retard SARS-CoV-2 transmission while minimising the impact on global citizens and the global economy? Are current infection control measures in prehospital and hospital settings sufficient and, if not, how can they be improved? We should note that the damage of COVID-19 has propagated far beyond the healthcare sector into almost all other industries. Furthermore, while conditions in some countries are beginning to ease, conditions in others are deteriorating. With our analysis of the early medical literature in the COVID-19 pandemic, we hope to unveil some of the critical gaps and longitudinal trends in the early-phase pandemic research and provide meaningful insight for future research and policy in both the present COVID-19 pandemic and future large-scale public health crises.

### Limitations

There are several limitations to our study. Firstly, our search period was restricted to a relatively early phase in the pandemic. Hence, the significance of our results in identifying specific gaps within the most recent literature was limited. However, these results from the early period of the pandemic are still able to provide a broad indication of the general focuses and trends within the research community and are thus relevant to research and policy priority setting for the COVID-19 pandemic and especially for future large-scale public health crises. Furthermore, we did not search databases such as Web of Science and Google Scholar. We acknowledge that exclusion of these databases reduces the comprehensiveness of our search. However, the use of two databases with the widest catchment of medical literature, PubMed and Embase, ensures that a sufficiently large proportion of the relevant literature is captured in our analysis. The focus of our study on general trends and gaps also did not necessitate an all-encompassing search. Hence, we used only two databases for this study to balance speed and accuracy given the need for an early indication of trends in this rapidly-evolving pandemic. We also did not search ClinicalTrials.gov, which would have excluded most clinical trial protocols. Also, only English language articles were analysed which resulted in the exclusion of articles from China — a substantial source of early COVID-19 literature. Lastly, the exclusion of non-peer reviewed research (those archived in medRxiv and bioRxiv) in our analysis may have neglected some new evidence but ensured the inclusion of only scientific results that have undergone peer review. Given the aforementioned pitfalls associated with the accelerated publication of COVID-19 articles even among peer-reviewed sources, we accepted the limitation of not capturing the latest research in order to ensure a baseline standard of reliability among the articles included for analysis.

## Conclusions

COVID-19 research is only in its nascent phase, but a large volume of research articles has already been published. An analysis of the early medical literature revealed valuable insights into the knowledge gaps that may remain unaddressed in current COVID-19 literature, as well as trends in the initial reaction of the research community that may be useful in research prioritisation and policy setting in future large-scale public health crises. Of particular concern and urgency are the mental health effects of the pandemic on health care workers, patients, and other populations. Other underexplored areas of research include the pathophysiology of the disease within different body systems and populations, the use of novel technologies such as artificial intelligence in forecasting trends and improving prevention and intervention strategies, and the indirect effects of the pandemic on the care of non-COVID-19 patients. International collaboration was also absent in most of the early literature. We opine that the systematic identification and filling of such gaps in the COVID-19 literature will be vital in informing and shaping effective, evidence-based governmental policies and healthcare practices, which can translate to improved outcomes in the present pandemic. Furthermore, the identified trends and gaps in the initial response of the research community to the COVID-19 pandemic will be valuable in guiding the priorities and actions of researchers, clinicians, and policymakers in the preparedness and response to future large-scale public health crises.

## Data Availability

The datasets used and/or analyzed during the current study are available from the corresponding author on reasonable request.

## Abbreviations

AI: Artificial intelligence
COVID-19: Coronavirus disease 2019
CT: Computed tomography
EGM: Evidence gap map
EHR: Electronic health record
IQR: Interquartile range
RCT: Randomised controlled trial
RT-PCR: Reverse transcriptase-polymerase chain reaction
USA: United States of America
WHO: World health organization
